# Preventive effects of quercetin against foot-and-mouth disease virus *in vitro* and *in vivo* by inducing type I interferon

**DOI:** 10.3389/fmicb.2023.1121830

**Published:** 2023-05-12

**Authors:** Gyeongmin Lee, Hyo Rin Kang, Aro Kim, Jong-Hyeon Park, Min Ja Lee, Su-Mi Kim

**Affiliations:** Center for Foot-and-Mouth Disease Vaccine Research, Animal and Plant Quarantine Agency, Gimcheon, Republic of Korea

**Keywords:** foot-and-mouth disease virus, antiviral effect, quercetin, interferon, FMD vaccine

## Abstract

Foot-and-mouth disease (FMD) is an acute contagious infectious disease that affects cloven-hoofed animals. Although current emergency FMD vaccines only take effect 7 days after vaccination, antiviral agents, such as quercetin, which is a common flavonoid, could reduce the spread of FMD virus (FMDV) during outbreaks. We investigated the *in vitro* and *in vivo* antiviral effects of quercetin against FMDV. Analysis of viral copy numbers showed that quercetin had a dose-dependent inhibitory effect on FMDV at concentrations between 19.5 and 1,250 μM in porcine cells. In addition, we observed a quercetin-induced interferon (IFN)-α protein and interferon-stimulated gene (ISG) upregulation in swine cells. Enzyme-linked immunosorbent assay of sera revealed that quercetin induces the production of IFN-α, IFN-β, IFN-γ, interleukin (IL)-12, and IL-15 in mice. Inoculation of mice with quercetin or a combination of quercetin with an inactivated FMD vaccine enhanced both the survival rate and neutralizing antibody titer. Therefore, we suggest the use of quercetin as a novel and effective antiviral agent for controlling FMDV infection; however, further investigation of its application in livestock is required.

## 1. Introduction

Foot-and-mouth disease (FMD) is an acute contagious infectious disease affecting cloven-hoofed animals, and the symptoms include fever, lameness, and vesicular eruptions of the mouth, tongue, snout, teats, and feet (Alexandersen et al., 2003). The FMD virus (FMDV) belongs to the genus *Aphthovirus* of the family Picornaviridae and consists of a single-stranded positive-sense RNA genome. FMDV has seven serotypes: A, O, C, Asia1, and South African territories 1, 2, and 3 (SAT1–3). The rapid spread of FMDV in susceptible animals renders FMD a severe disease as listed by the World Organization for Animal Health (WOAH). However, emergency FMD vaccines that induce early protection have limited usage because a minimum of 7 days following the administration of the vaccine in pigs is required to realize its effects (Salt et al., 1998). Therefore, an alternative method of administering antiviral agents is necessary to reduce the spread of FMDV during outbreaks. Interferons (IFNs) or IFN stimulators have been reported to show antiviral effect against FMDV (Moraes et al., 2007; Kim et al., 2015; Diaz-San Segundo et al., 2021; Lee et al., 2022). Ribavirin, 6-azauridine, and guanidine-HCl have been reported as antiviral agents that inhibit FMDV replication by lethal mutagenesis or by blocking viral proteins (Nettleton et al., 1982; Airaksinen et al., 2003; Kim et al., 2012; Sadeghipour et al., 2012).

Quercetin is a common flavonoid in plant-based foods and beverages and displays antioxidant, immunomodulatory, anticancer, and antiviral properties (Kaul et al., 1985; Hollman et al., 1997; Patil et al., 2009; Tanaka et al., 2020). In particular, its antiviral effects have been reported in poliovirus, respiratory syncytial virus, human hepatitis C virus, enterovirus 71 (EV71), and porcine epidemic diarrhea virus (Kaul et al., 1985; Choi et al., 2009; Rojas et al., 2016; Yao et al., 2018). Recently, quercetin has been proposed as an antiviral drug against severe acute respiratory syndrome coronavirus 2 (SARS-CoV-2) (Derosa et al., 2021). It may influence viral replication via the regulation of viral or host protein expression (Rojas et al., 2016; Yao et al., 2018; Derosa et al., 2021). Quercetin blocks EV71 3C*^pro^* and reduces endocytosis of human rhinovirus (Ganesan et al., 2012; Yao et al., 2018). Previous studies tested the therapeutic effect of quercetin against FMDV in cells. Although other flavonoids, such as luteolin and isoginkgetin, were proven to be the 3C*^pro^* of FMDV inhibitors, the therapeutic effect of quercetin was tested and a significant inhibitory effect against FMDV was not observed (Qian et al., 2015; Theerawatanasirikul et al., 2021). However, the prophylactic application of antiviral agents against FMDV is more robust compared with the therapeutic application because FMD is not controlled by treating individual animals but rather by protecting against viral infection and transmission. In addition, the effective treatment methods of antiviral agents could be different depending on their antiviral mechanisms. The adjuvant effect of quercetin based on its combination with ovalbumin antigen has been reported in mice (Singh et al., 2017). Therefore, studies on the prophylactic effect of quercetin or the combination of quercetin with vaccines against FMDV is needed.

Foot-and-mouth disease virus is highly sensitive to interferon-α (IFN-α), which has been reported to enhance humoral and cell-mediated immunity (Chinsangaram et al., 2001; Le Bon et al., 2001; Moraes et al., 2007; Gujer et al., 2011), Therefore, the type I IFN like IFN-α is a promising antiviral candidate for a combination strategy with an inactivated FMD vaccine (Kim et al., 2015; Diaz-San Segundo et al., 2016; You et al., 2017). Although quercetin induces the expression of type I IFN in cancer cells (Tai et al., 2012; Igbe et al., 2017; Peng et al., 2020), verification that this process constitutes an antiviral mechanism is required. Therefore, whether quercetin induces type I IFN in normal cells or animals and has prophylactic effects against FMDV should be further tested.

In this study, we tested the prophylactic effects of quercetin against FMDV *in vitro* and *in vivo* by evaluating the inhibition properties of quercetin on FMDV replication in swine cells and mice. In addition, we analyzed the antiviral mechanisms of quercetin by testing type I IFN activation in swine cells and demonstrating cytokine induction in mice. Furthermore, we investigated the antiviral and adjuvant effects of quercetin in combination with FMD-inactivated vaccine in mice.

## 2. Materials and methods

### 2.1. Reagents, cells, and viruses

Commercial quercetin and ribavirin (≥95%) were purchased from Sigma-Aldrich (St. Louis, MO, USA). Stock solutions of quercetin dissolved in dimethyl sulfoxide and ribavirin dissolved in water were diluted in phosphate-buffered saline (PBS) or Dulbecco’s modified Eagle’s medium (DMEM; Thermo Fisher Scientific, Waltham, MA, USA) for experiments. Porcine kidney (LFBK) cells, which were supplied by the Plum Island Animal Disease Center (Orient, NY, USA), were cultured in DMEM (Thermo Fisher Scientific, Waltham, MA, USA) supplemented with 10% fetal bovine serum (pH 7.4), and incubated at 37^°^C in an atmosphere of 5% CO_2_. The FMDV serotype O, O/SKR/Boeun/2017 (GenBank accession number: MG983730) was used to infect LFBK cells and FMDV serotype O, O/VIT/2013 adapted to mice was used in *in vivo* experiments as described previously (Lee et al., 2022). Viral titers were calculated using the Spearman-Karber method at 50% tissue culture infective dose (TCID_50_) (Ramakrishnan, 2016). All *in vitro* experiments employing FMDV were conducted in a bio-safety level 3 facility at the Animal and Plant Quarantine Agency (APQA), Republic of Korea.

### 2.2. FMDV inhibition assay and cytotoxicity assay in swine cells

This test was performed as previously described (Kim et al., 2012; Lee et al., 2022). The inhibitory effects on FMDV and cytotoxicity of the antiviral agents were measured in LFBK cells. LFBK cells (2 × 10^4^ cells/well) were seeded in 96-well plates. The following day, the cells were treated with serially diluted quercetin or ribavirin (positive control). The cells were infected with O/SKR/Boeun/2017 at a 0.001 multiplicity of infection (MOI) and the inoculum was replaced with new media 2 h post FMDV infection. Following incubation at 37^°^C for 24 h, the viability of infected cells was determined using the 3-(4,5-dimethylthiazol-2-yl)-5-(3-carboxymethoxyphenyl)-2-(4-sulfophenyl)-2H-tetrazolium (MTS) assay using a CellTiter 96 AQueous One Solution Cell Proliferation Assay Kit (Promega, Madison, WI, USA). Antiviral activity was expressed as the 50% effective concentration (EC_50_), defined as the concentration that reduces virus-induced cytopathogenicity by 50% of the control value. Cytotoxicity was expressed as the 50% cytotoxic concentration (CC_50_), defined as the concentration required to reduce cell viability by 50% of the control value and determined using the MTS assay. For the cytotoxicity assay, cultured cells were treated with antiviral agents without FMDV infection.

### 2.3. Dose-dependent antiviral effect of quercetin in swine cells assay

LFBK cells (3.5 × 10^4^ cells/well) were seeded in 96-well plates. The following day, the cells were treated with serially diluted quercetin or ribavirin. After removing the inoculum, they were infected with FMDV O/SKR/Boeun/2017 at 0.001 MOI at 24 h post-treatment. The inoculum was replaced with a new medium 2 h post-infection and the cells were incubated at 37^°^C. Then, the supernatants were collected 24 h post FMDV infection. To evaluate FMDV RNA replication, we performed RNA extraction and quantitative real-time reverse transcription-PCR (RT-qPCR) (Kim et al., 2010).

### 2.4. Time-of-addition assay

This test was performed as described previously (Lee et al., 2022). LFBK cells (3.5 × 10^4^ cells/well) were seeded in 96-well plates. The following day, the cells were treated with 625 μM quercetin 2 h before FMDV O/SKR/Boeun/2017 infection (Pre-2 h), at the time of infection (Co-0 h), 2 h post-infection (Post-2 h), or 4 h post-infection (Post-4 h). The cells were infected with FMDV at 0.001 MOI for 2 h and the medium was changed thereafter. At each step, all cells were washed twice with DMEM and the control group was only infected with FMDV. The cells were incubated at 37^°^C and the supernatants were collected 24 h post FMDV infection. Viral titration was performed using the supernatant as mentioned above.

### 2.5. IFN-α induction by quercetin in swine cell assay

LFBK cells (6 × 10^4^ cells/well) were seeded in 24-well plates. The following day, the cells were treated with 625 μM quercetin or left untreated (cell control) and incubated at 37^°^C. After replacing the medium with fresh medium at 24 h post-treatment, the cells were incubated at 37^°^C for 24 or 48 h. The supernatant was collected at 48 or 72 h post-treatment and the quantity of porcine IFN-α protein in the supernatant was determined using enzyme-linked immunosorbent assay (ELISA) (Abcam, Cambridge, United Kingdom).

### 2.6. Evaluating quercetin-induced upregulation of interferon and interferon-stimulated genes (ISGs) in swine cells

LFBK cells (4 × 10^5^ cells/well) seeded in 6-well plates were treated with 625 μM quercetin and collected at 6, 18, and 24 h post-treatment. RNA extraction, DNase I treatment, and RT-qPCR were performed as described previously (Lee et al., 2022). RT-qPCR was used to IFN-α, IFN-β, determine 2′–5′ oligoadenylate synthetase (*OAS*), protein kinase R (*PKR*), and myxovirus resistance (*Mx*) expression levels. Porcine glyceraldehyde 3-phosphate dehydrogenase (*GAPDH*) was used as the internal control. Primers or dual-labeled TaqMan probes (5′FAM/3′BHQ-1) were designed as described previously (Moraes et al., 2007; Li et al., 2017; Supplementary Table 1) and synthesized by Bioneer Corporation (Daejeon, Republic of Korea).

### 2.7. Cytokine assay in mice

A total of 7 weeks-old female BALB/c mice were purchased from Cosa-Bio (Seongnam, Republic of Korea). The study protocol was approved by the Animal Care and Use Committee of the APQA in the Republic of Korea (approval number: 2021-579). All *in vivo* experiments were conducted in an animal bio-safety level 3 facility at the APQA. The mice were intraperitoneally injected with 625 μM quercetin (0.1 mL). Blood was collected at 0, 6, 16, and 24 h post-inoculation (four mice per group), and corresponding sera were analyzed to evaluate the concentration of IFN-α, IFN-β, IFN-γ, IL-12, IL-15, and IL-6 using mouse IFN-α, IFN-β, IFN-γ, IL-12, IL-15 (MyBioSource, San Diego, CA, USA), and IL-6 ELISA kits (Thermo Fisher Scientific, Waltham, MA, USA). Protein concentrations were determined by the interpolation of standard curves.

### 2.8. Measurement of antiviral effects in mice

To evaluate the effects of quercetin with respect to FMDV, 7 weeks-old female C57BL/6 mice were intraperitoneally injected with 0.1 mL PBS (negative control group), 25 or 50 mg/kg of ribavirin (positive control group) (Gu et al., 2006), or 25 or 50 mg/kg of quercetin at 16 h, 1 day, or 3 days prior to FMDV infection. The mice were intraperitoneally injected with 0.1 mL mouse-adapted FMDV O/VIT/2013 (250 LD_50_) (Ko et al., 2019). All mice (five per group) were observed for 7 days post-infection (DPI).

### 2.9. Combined effect of quercetin with the FMD vaccine and virus neutralization test

To evaluate the antiviral effect of quercetin in combination with a whole virus based FMD inactivated vaccine, mice were intramuscularly injected with 0.1 mL PBS (non-vaccinated group), FMD vaccine, or a combination of quercetin and vaccine 1 or 3 days prior to FMDV infection. The vaccine was formulated using the O/SKR/Boeun/2017 vaccine antigen (0.1 μg/dose), 10% aluminum hydroxide gel (Rehydragel HPA; General Chemical, Moorestown, NJ, USA), and Montanide ISA 206 (Seppic, Paris, France) in a water-in-oil-in-water emulsion (Lee et al., 2020; Hwang et al., 2021). The mixture of the FMD vaccine and quercetin was prepared by vortexing. The mice were intraperitoneally injected with 0.1 mL mouse-adapted FMDV O/VIT/2013 (250 LD_50_) (Ko et al., 2019). All mice (five per group) were observed for 7 DPI. To determine the adjuvant effect of quercetin in combination with an FMD vaccine, mice were intramuscularly injected with 0.1 mL FMD vaccine formulated using O/SKR/Boeun/2017 vaccine antigen (1 μg/dose), 10% aluminum hydroxide gel, and Montanide ISA 206, or a combination of quercetin and the FMD vaccine. Blood samples were collected at 1, 2, 3, and 4 weeks post-vaccination (WPV). Serum was separated from the samples and heat-inactivated at 56^°^C for 30 min. The virus neutralizing antibody test (VNT) was performed according to the guidelines of the Manual of Diagnostic Tests and Vaccines for Terrestrial Animals (WOAH, 2022). FMDV O/SKR/Boeun/2017, passaged four times in LFBK cells, was used for the VNT. A neutralization reaction was performed between serially diluted sera and 100 TCID_50_ FMDV at 37^°^C for 1 h. Then, the neutralized viruses were placed on 96-well microplates and LFBK cells were added. The microplates were incubated at 37^°^C for 48–72 h to assess the CPE. Neutralizing antibody titers were calculated as the reciprocal number of the maximum serum dilution that neutralized 100 TCID_50_ FMDV using the Spearman-Karber method.

### 2.10. Statistical analysis

Unpaired *t*-tests and the evaluation of EC_50_ and CC_50_ were performed using GraphPad Prism v5.0 (La Jolla, CA, USA). Significance was set at *P* < 0.05.

## 3. Results

### 3.1. *In vitro* antiviral effect of quercetin

#### 3.1.1. Selectivity of quercetin as a FMDV inhibitor

Inhibition of the FMDV-induced cytopathic effect and cytotoxicity of antiviral agents were determined in swine cells (Table 1). The selectivity index of agents was calculated as the ratio of CC_50_ to EC_50_. Quercetin and ribavirin possessed high selectivity indices of 138.79 and 117.67, respectively. Importantly, quercetin displayed a higher selectivity index and lower cytotoxicity than ribavirin, a well-known anti-FMDV agent.

**TABLE 1:** Antiviral effect of quercetin against FMDV.

Compound	CC_50_^a^	EC_50_^b^	SI^c^
Quercetin	927.05 ± 56.56 μM^d^	6.68 ± 0.50 μM	138.79
Ribavirin	879.25 ± 36.98 μM	7.47 ± 0.15 μM	117.67

^a^Cytotoxic concentration required to reduce cell viability by 50% of the control value.

^b^Effective concentration required to reduce virus-induced cytopathogenicity by 50% of the control value.

^c^Selectivity index = CC_50_/EC_50_.

^d^Data are presented as the mean ± standard deviation (SD) of three independent experiments.

#### 3.1.2. Dose-dependent FMDV inhibition in swine cells

**FIGURE 1:**
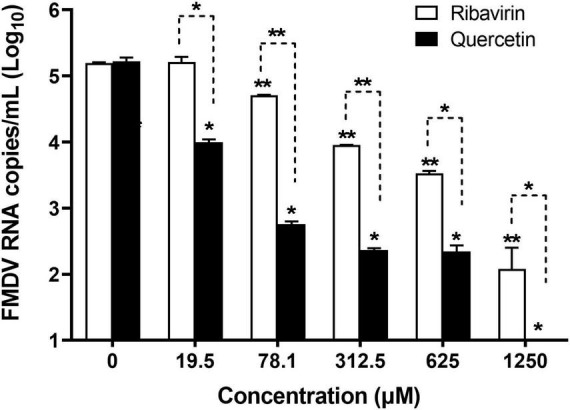
Dose-dependent anti-foot-and-mouth disease virus (FMDV) effect of quercetin in swine cells. LFBK cells were treated with a series of quercetin or ribavirin (positive control) dilutions and infected with FMDV at 0.001 MOI. The supernatant was collected 24 h post-infection and viral copy numbers were measured using quantitative real-time reverse transcription-PCR (RT-qPCR). Error bars indicate standard deviations from the mean. Unpaired *t*-tests were performed to identify significant differences. *P < 0.05, **P < 0.01.

Viral copy numbers were analyzed in FMDV-infected LFBK cells treated with serially diluted quercetin or ribavirin (positive control) (Figure 1). Compared to untreated cells infected with FMDV, those treated with quercetin showed an antiviral effect at concentrations of 19.5–1,250 μM (*P* < 0.05), the viral copy numbers decreased in a dose-dependent manner (*P* < 0.05). Cells treated with 78.1–1,250 μM ribavirin displayed a similar dose-dependent reduction in FMDV copy numbers. Viral replication was significantly lower in cells treated with quercetin when compared to cells treated with ribavirin (*P* < 0.05).

#### 3.1.3. Prophylactic and therapeutic effect against FMDV

**FIGURE 2:**
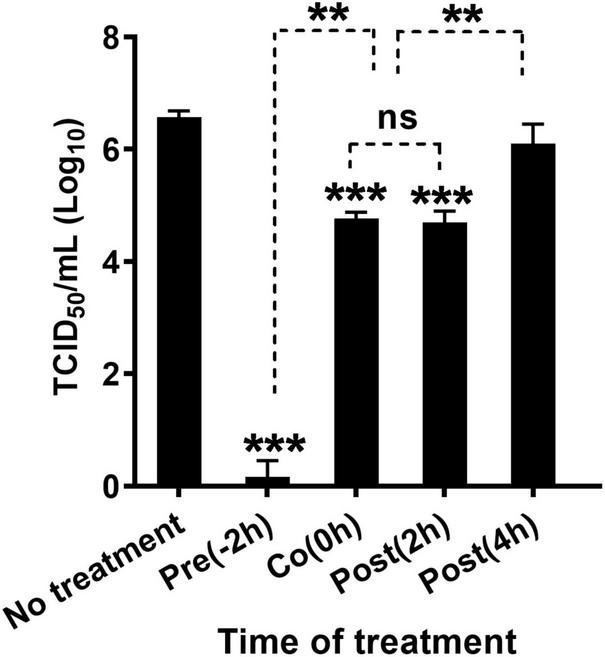
Time-of-addition experiment investigating the viral stage at which quercetin is effective. LFBK cells were treated with 625 μM quercetin 2 h prior to foot-and-mouth disease virus (FMDV) infection (Pre-2 h), at the time of infection (Co-0 h), at 2 h (Post-2 h), or 4 h (Post-4 h) post-infection; cells were infected at 0.001 MOI for 2 h. In each step, cells were washed twice using Dulbecco’s modified Eagle’s medium (DMEM). The supernatant was collected 24 h post-infection, and virus titration was performed. Error bars indicate standard deviations from the mean. Unpaired *t*-tests were performed to identify significant differences. **P < 0.01, ***P < 0.005, ns, not significant.

To explore how quercetin targets the FMDV replication cycle, a time-of-addition test was performed (Figure 2). Reduced FMDV titers were observed in Pre-2 h, Co-0 h, and Post-2 h cells treated with quercetin (*P* < 0.005). However, viral replication significantly reduced in the case of Pre-2 h cells when compared to the untreated Co-0 h, Post-2 h, and Post-4 h cells (*P* < 0.01). In addition, the antiviral effect of quercetin was noted during virus entry (Co-0 h) and the early stage (Post-2 h), but not the late stage (Post-4 h), of the replication cycle. Viral replication showed no significant difference between Co-0 h and Post-2 h cells after quercetin treatment.

### 3.2. Induction of porcine IFN-α and activation of ISGs in swine cells

**FIGURE 3:**
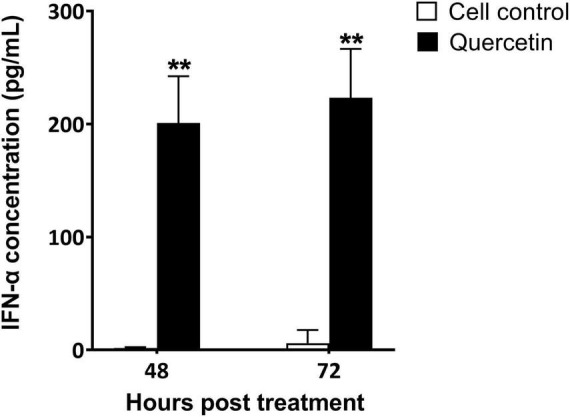
Interferon (IFN)-α induction by quercetin in swine cells. LFBK cells were treated with 625 μM quercetin for 24 h and then supplied with new medium. The supernatant was collected 48 or 72 h post-treatment and the quantity of IFN-α was measured using enzyme-linked immunosorbent assay (ELISA). Error bars indicate standard deviations from the mean. Unpaired *t*-tests were performed to identify significant differences. **P < 0.01.

**FIGURE 4:**
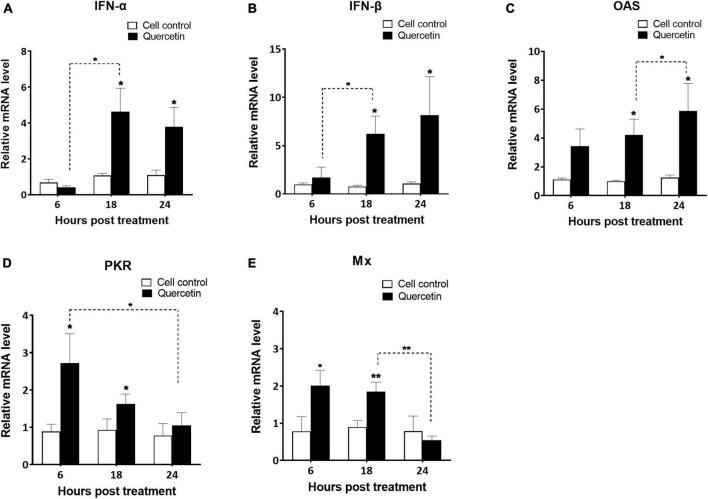
Activation of interferon-stimulated genes in swine cells treated with quercetin. LFBK cells were treated with 625 μM quercetin, collected after 6, 18, and 24 h, and mRNA levels of interferon-stimulated genes such as IFN-α **(A)**, IFN-β **(B)**, OAS **(C)**, PKR **(D)**, and Mx **(E)** were analyzed using quantitative real-time reverse transcription-PCR (RT-qPCR). Error bars indicate standard deviations from the mean. Unpaired *t*-tests were performed to identify significant differences. *P < 0.05, **P < 0.01.

The supernatant of swine cells treated with quercetin displayed higher levels of porcine IFN-α than that of untreated cells in both 48 and 72 h post-treatment (Figure 3) (*P* < 0.01). Interferon-stimulated gene (ISG)s such as *IFN-*α, *IFN-*β, *OAS*, *PKR*, and *Mx* were significantly upregulated in quercetin-treated cells when compared to controls (*P* < 0.05). Significantly enhanced transcript-level expression of *IFN-*α, *IFN-*β, and *OAS* was detected at 18 and 24 HPT (Figures 4A–C, *P* < 0.05). The transcript-level expression of *IFN-*α and *IFN-*β increased from 6 to 18 HPT (*P* < 0.05) and was maintained at 24 HPT. Furthermore, the transcript-level expression of *OAS* increased from 18 to 24 HPT (*P* < 0.05). Transcript-level expression of *PKR* and *Mx* was upregulated at 6 and 18 HPT (*P* < 0.05), and the transcription level decreased from 6 to 24 HPT (Figures 4D, E, *P* < 0.05).

### 3.3. Cytokine activation in mice

**FIGURE 5:**
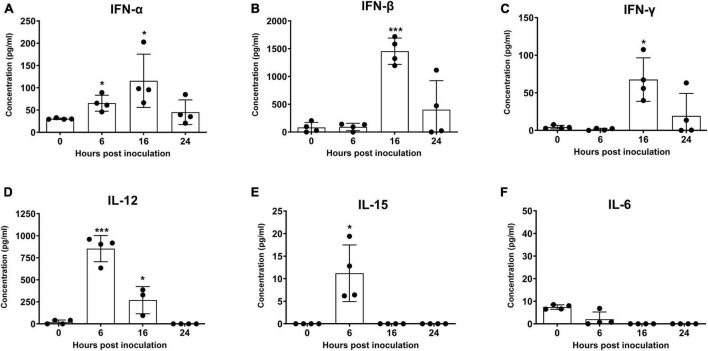
Induction of cytokines by quercetin in mice. BALB/c mice were inoculated with 50 mg/kg quercetin through intraperitoneal injection. Sera were collected 0, 6, 16, and 24 h post-treatment. Protein levels of murine interferon IFN-α **(A)**, IFN-β **(B)**, IFN-γ **(C)**, interleukin (IL)-12 **(D)**, IL-15 **(E)**, and IL-6 **(F)** were measured using enzyme-linked immunosorbent assay (ELISA). Error bars indicate standard deviations from the mean. Unpaired *t*-tests were performed to identify significant differences. *P < 0.05, ***P < 0.005.

Following inoculation of mice with quercetin, IFN-α, IFN-β, IFN-γ, IL-12, and IL-15 levels were significantly increased at 6, 16, or 24 h (Figure 5, *P* < 0.05). The highest levels of IFN-α, IFN-β, and IFN-γ were recorded at 16 h, and reduced at 24 h (Figures 5A–C, *P* < 0.05). However, the levels of IL-12 and IL-15 were the highest at 6 h but dropped again at 16 h (Figures 5D, E, *P* < 0.05). Enhancement of IL-6 was not detected in mice inoculated with quercetin (Figure 5F).

### 3.4. Enhanced survival rate of mice treated with quercetin

**FIGURE 6:**
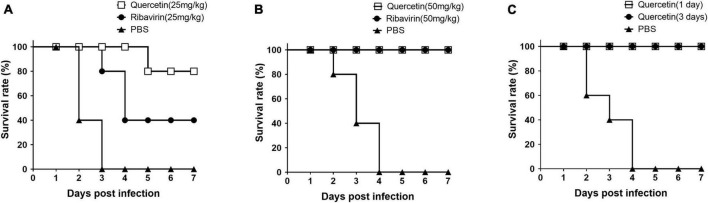
Survival rate of mice inoculated with quercetin and infected with foot-and-mouth disease virus (FMDV). C57BL/6 mice were inoculated with 25 mg/kg **(A)** or 50 mg/kg **(B,C)** of either quercetin or ribavirin (positive control) through intraperitoneal (IP) injection. After 16 h **(A,B)**, 1 or 3 days **(C)**, all mice were infected with a 250 LD_50_ of FMDV (O/VIT/2013) through intraperitoneal (IP) injection. Mice with phosphate-buffered saline (PBS) inoculation served as a negative control. The mice were monitored for 7 days.

To test the inhibition effect of quercetin on FMDV replication *in vivo*, mice were inoculated with quercetin or ribavirin and subsequently infected with FMDV at 16 h post-treatment (Figure 6). The group treated with quercetin or ribavirin had an improved survival rate when compared to the mice in control group with a treatment of 25 mg/kg (Figure 6A). However, the survival rate of mice receiving quercetin (80% at 7 DPI) was higher than that of mice receiving ribavirin (40% at 7 DPI). Upon doubling the dosage to 50 mg/kg, the murine survival rate improved further; mice receiving either quercetin or ribavirin showed 100% survival rate in the background of FMDV infection at 7 DPI (Figure 6B). The 100% survival rate up to 7 DPI was maintained in mice inoculated with quercetin, either 1 or 3 days prior to FMDV exposure (Figure 6C).

### 3.5. Antiviral and adjuvant quercetin with FMD vaccine in mice

**FIGURE 7:**
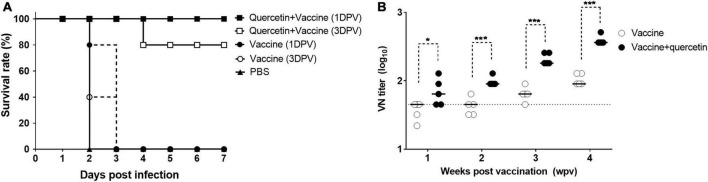
Prevention effect of quercetin combined with inactivated foot-and-mouth disease (FMD) vaccine. C57BL/6 mice were inoculated via intramuscular (IM) injection with a mixture of 50 mg/kg of quercetin and inactivated FMD vaccine or with FMD vaccine. The FMD vaccine was formulated using Montanide ISA 206 (Seppic, France), 10% aluminum hydroxide gel, and 0.1 μg **(A)** or 1 μg **(B)** of O/SKR/Boeun/2017 antigen. **(A)** Vaccinations were performed 1 or 3 days before FMDV infection and phosphate-buffered saline (PBS) was introduced 1 day before foot-and-mouth disease virus (FMDV) challenge as the negative control. All mice were infected with a 250 LD_50_ of FMDV (O/VIT/2013) through intraperitoneal (IP) injection and monitored for 7 days. **(B)** Serum samples were collected at 1, 2, 3, and 4 weeks post-vaccination. The virus neutralizing antibody test was performed using serum samples. The dotted line indicates the 1:45 (log_10_ 1.65) viral neutralizing titer cut-off level. Error bars indicate standard deviations (SDs) from the mean. Unpaired *t*-tests were performed to identify statistically significant differences between the vaccine group and others or among the combination groups (*P < 0.05; ***P < 0.005).

The survival rate of mice receiving only the inactivated FMD vaccine was significantly lower (0% at 7 DPI) (Figure 7). However, a survival rate of 80–100% at 7 DPI was maintained in all mice that intramuscularly received a combination of quercetin and inactivated FMD vaccine, either 1 or 3 days prior to FMDV infection (Figure 7A). Furthermore, virus neutralizing antibody titers were higher at 1-, 2-, 3- and 4 weeks post-vaccination (WPV) in mice in the combination group than in mice in the vaccine group (*P* < 0.05, Figure 7B).

## 4. Discussion

The antiviral agents could promptly block spreading of FMDV. The combination of FMD vaccine and anti-FMDV agents, which can be used for prophylactic purposes, is ideal. We found quercetin to be an effective treatment prior to FMDV infection, at the viral entry stage, and during early stages of viral replication. Importantly, quercetin may induce type I IFN expression, thereby activating ISGs to create an antiviral environment before the actual infection (Schneider et al., 2014). FMDV replicates rapidly, and its progeny can be observed 5 h post-infection (Kumar et al., 2016). Therefore, quercetin administration could provide an appropriate preventive strategy against FMDV infection. In addition, antivirals may help overcome the immune gap associated with FMD emergency vaccines. Quercetin in combination with an inactivated FMD vaccine that is commonly used to control outbreaks was effective, thus supporting its use as a promising strategy for the early protection of animals against FMDV. In a previous study, Qian et al. (2015) tested the antiviral effect of quercetin against FMDV for the therapeutic application to cells but did not observe a significant effect. However, we proved the significant antiviral effect of quercetin or the combination of quercetin and FMD vaccine for the preventive application in porcine cells and animals. The induction of type I IFN could be an effective antiviral mechanism for the preventive treatment. In addition, we tested the antiviral effects and type I IFN induction of quercetin in porcine cells, whereas Qian et al. (2015) used baby hamster kidney (BHK)-21 cells, known to be deficient in type I IFN production (MacDonald et al., 2007).

We suggest that induction of type I IFN expression could be the decisive mechanism by which quercetin inhibits FMDV. Quercetin induced porcine IFN-α production and activated ISG mRNA expression in swine cells; porcine IFN-α is known to be a highly effective antiviral agent against FMDV (Moraes et al., 2007; Dias et al., 2012; Kim et al., 2014). The antiviral effect of quercetin pretreatment was most remarkable during the time of addition test. The induction of type I IFN expression by quercetin in cancer therapy has been reported in cancer cells (Tai et al., 2012; Igbe et al., 2017; Peng et al., 2020). Although different factors, such as retinoic acid-inducible gene I (RIG-I) and Src homology 2 domain-containing protein tyrosine phosphatase-2 (SHP-2) are related to the induction of IFN expression, quercetin has been reported to enhance IFN-α expression and activate Janus kinase/signal transducers and activators of transcription (JAK/STAT) signaling in cancer cells. Therefore, it is necessary to identify genes regulating quercetin-induced IFN stimulation in healthy cells in further studies. In addition, the pan-antiviral effects of quercetin and its derivates are well-known and the various mechanisms have been described (Di Petrillo et al., 2022). Therefore, whether the antiviral mechanisms of quercetin against various viruses are related to the induction of type I IFN must be clarified in further studies.

The essential protein in viral replication and one of the most conserved regions in viral genes, 3C pro is an attractive target for antiviral drugs against picornaviruses, including FMDV (Sun et al., 2016). Quercetin was known as the inhibitor of 3C pro of Enterovirus 71 (Yao et al., 2018). Luteolin and isoginkgetin, which are well-known flavonoids, had antiviral effects on FMDV *in vitro* as 3C protease inhibitors (Theerawatanasirikul et al., 2021). However, quercetin 7-rhamnoside did not show binding affinity to FMDV 3C pro in the study. In this study, quercetin inoculation was also more effective at early stages of viral replication than at the late stage of FMDV replication. Therefore, we cannot exclude the possibility that quercetin has a binding affinity toward 3C pro FMDV. The β-ribbon of 3C pro in FMDV binds to and effectively cleaves the VP1/2A peptide (APAKE/LLNFD) (Birtley et al., 2005; Sweeney et al., 2007; Zunszain et al., 2010). In the simulation using a molecular docking model, we observed that the region of quercetin involved in binding to 3C pro was near the β-ribbon, overlapping with the region of VP2/2A involved in binding to 3C pro (Supplementary Figures 1A, B). Furthermore, three amino acids of 3C pro (Gly-161, Ala-163, and Gly-184) formed hydrogen bonds with either VP1/2A or quercetin (Supplementary Figures 1C, D). Additionally, we observed a hydrogen bond between the His-46 residue of 3C pro and quercetin. His-46 is known to be one of the critical residues of the catalytic triad located at the center of the peptide-binding cleft of 3C pro (Curry et al., 2007). Flavonoids such as luteolin and isoginkgetin, which are potential 3C pro inhibitors, formed similar hydrogen bonds with the His-46 or Cys-163 residues of 3C pro (Theerawatanasirikul et al., 2021). Although the main mechanism of antiviral effect of quercetin must be induction of type I IFN, the unclear mechanism of quercetin as a 3C pro inhibitor requires further investigation.

We showed that the antiviral and adjuvant effects of quercetin could be strengthened by the induction of type I IFN. Type I IFN inhibits viral replication and stimulates dendritic, T and B cells (Le Bon et al., 2001; Le Bon et al., 2006). Specially, IFN-α has been reported as an effective adjuvant for FMD vaccines (Cheng et al., 2007; Su et al., 2013). Furthermore, we observed that quercetin induced the production of IFN-γ, IL-12, and IL-15, which are related to B and T-cell immunity and have an inhibitory effect on viral replication (Armitage et al., 1995; Carr et al., 1997; Sartori et al., 1997; Tewari et al., 2007; Verbist and Klonowski, 2012). IL-12 and IL-15 stimulate IFN-γ production and activate natural killer (NK) cells (Fehniger et al., 1999; Xu et al., 2010; de Groen et al., 2015; Konjević et al., 2019). The antiviral effects of IFN-γ and IL-12 against FMDV were previous reported (Moraes et al., 2007; Toka et al., 2009; Diaz-San Segundo et al., 2010). Quercetin induces the expression of the T helper type 1 (Th1) cell-derived cytokine IFN-γ and regulates the Th1/T helper type 2 (Th2) balance (Park et al., 2009; Chirumbolo, 2010; Tanaka et al., 2020). However, quercetin did not induce IL-6 expression or Th2 cell immunity and lowered the levels of IL-6 induced in response to lipopolysaccharide treatment (Kandere-Grzybowska et al., 2006; Huang et al., 2010; Lee et al., 2016). We also did not observe the induction of IL-6 expression by quercetin *in vivo*. It could be good for the safety of animals since IL-6 is associated with allergic asthma or cytokine storms (Doganci et al., 2005; Chen et al., 2020).

The safety, effects, and pharmacodynamics are important factors to consider when determining the dose of quercetin *in vivo*. We observed a dose-dependent antiviral effect against FMDV *in vitro* at 19.5–1,250 μM and *in vivo* at 25 and 50 mg/kg. Since few *in vivo* studies have reported on the antiviral effects of quercetin, we designed the experiments by the modification of anti-inflammation studies on quercetin (Veckenstedt and Pusztai, 1981; Veckenstedt et al., 1987; Ganesan et al., 2012). The effective doses of quercetin that provided an antiviral effect against cardiovirus and immunoglobulin E (IgE) inhibition in mice were 20 or 25 mg/kg (Veckenstedt and Pusztai, 1981; Okumo et al., 2021). We observed that these doses (25 and 50 mg/kg) did not decrease the survival rate or body weight of mice (data not shown). In humans, a daily oral dose of 1,000 mg of the quercetin derivative did not induce safety concerns and had a therapeutic effect in COVID-19 patients (Di Pierro et al., 2021). Although quercetin is considered safe, its low solubility in water and low absorption rate in the body serve as limitations for its clinical application in humans (Massi et al., 2017). Therefore, novel derivatives or delivery methods have been investigated (Jain et al., 2013; Liu et al., 2019; Alizadeh and Ebrahimzadeh, 2022). We used an intramuscular injection to administer a combination of quercetin and ISA 206 adjuvant vaccine, and observed effective antiviral effects in mice. In previous studies, quercetin has also been administered using water-in-oil-in-water double emulsions or nanoparticles, thereby enhancing the duration of effect and delivery efficiency (Lotfi et al., 2021; Banik et al., 2022; Han et al., 2022). Approaches to determine the pharmacodynamics and safety of intramuscularly injected quercetin are needed to determine the optimal dose for livestock because these factors could change according to species and injection routes.

In conclusion, our study demonstrates that quercetin, a safe and inexpensive material, is a promising antiviral agent against FMDV. Furthermore, we observed adjuvant and early protection effects of quercetin based on its combination with the FMD vaccine. Future studies can employ modified quercetin with enhanced solubility in natural hosts, such as pigs, and further evaluate the therapeutic effects of quercetin with an oil adjuvant FMD vaccine.

## Data availability statement

The original contributions presented in this study are included in the article/Supplementary material, further inquiries can be directed to the corresponding author.

## Ethics statement

This animal study was reviewed and approved by Animal and Plant Quarantine Agency.

## Author contributions

S-MK designed the study. GL, HK, and AK performed the experiments. S-MK and GL analyzed the data and wrote the original draft. J-HP and ML reviewed the draft. All authors read and approved the final manuscript.

## Supplementary material

Supplementary Figure 1 Molecular docking prediction for evaluating the interaction between foot-and-mouth disease virus (FMDV) 3C protease and quercetin. Surface representation of the binding pocket of 3C protease (PDB ID: 2WV5) (gray) with either **(A)** the FMDV VP1/2A peptide (cyan) or **(B)** quercetin (CID: 5280343) (green), relative to the β-ribbon of 3C pro (orange), respectively. An illustration of hydrogen bond formation between FMDV 3C pro and either the FMDV VP1/2A peptide **(C)** or quercetin **(D)**, depicting hydrogen bonds (dotted red lines), VP1/2A peptide residues (cyan sticks), 3C pro residues (black sticks), and quercetin residues (green sticks). Amino acids in bold are residues forming hydrogen bonds with both **(C,D)**.
