# Supplementary material for: Preventive effects of quercetin against foot-and-mouth disease virus in vitro and in vivo by inducing type I interferon
Source: Front Microbiol. 2023 May 12;14:1121830. doi: 10.3389/fmicb.2023.1121830 (PMC10213290; doi:10.3389/fmicb.2023.1121830)
Supplement: Supplementary file 1 [file Table_1.docx]

Supplementary table 1. Primers and probes used for detecting ISGs in this study.

| Gene | Primer/Probes | Sequence 5′ to 3′ | Reference |
| --- | --- | --- | --- |
| IFN-α | Forward primer | TGGTGCATGAGATGCTCCA | Moraes et al., 2007 |
|  | Reverse primer | GCCGAGCCCTCTGTGCT |  |
|  | Probe (5ʹFAM/3ʹBHQ-1) | CAGACCTTCCAGCTCT |  |
| IFN-β | Forward primer | AGTGCATCCTCCAAATCGCT |  |
|  | Reverse primer | GCTCATGGAAAGAGCTGTGG |  |
|  | Probe (5ʹFAM/3ʹBHQ-1) | TCCTGATGTGTTTCTC |  |
| Mx | Forward primer | GAGGTGGACCCCGAAGGA |  |
|  | Reverse primer | CACCAGATCCGGCTTCGT |  |
|  | Probe (5ʹFAM/3ʹBHQ-1) | AGGACCATCGGGATC |  |
| OAS | Forward primer | CTGTCGTTGGACGATGTATGCT |  |
|  | Reverse primer | CAGCCGGGTCCAGAATCA |  |
|  | Probe (5ʹFAM/3ʹBHQ-1) | TCAAGAAACCCAGGCCT |  |
| GAPDH | Forward primer | CGTCCCTGAGACACGATGGT |  |
|  | Reverse primer | CCCGATGCGGCCAAAT |  |
|  | Probe (5ʹFAM/3ʹBHQ-1) | AAGGTCGGAGTGAACG |  |
| PKR | Forward primer | GGAAGAAAACAAACACAGCTTGAA | Li et al., 2017 |
|  | Reverse primer | CCAAATCCACCTGAGCCAATT |  |
| GAPDH | Forward primer | ACATGGCCTCCAAGGAGTAAGA |  |
|  | Reverse primer | GATCGAGTTGGGGCTGTGACT |  |
