# Supplementary figures and images for: Preventive effects of quercetin against foot-and-mouth disease virus in vitro and in vivo by inducing type I interferon
Source: Front Microbiol. 2023 May 12;14:1121830. doi: 10.3389/fmicb.2023.1121830 (PMC10213290; doi:10.3389/fmicb.2023.1121830)

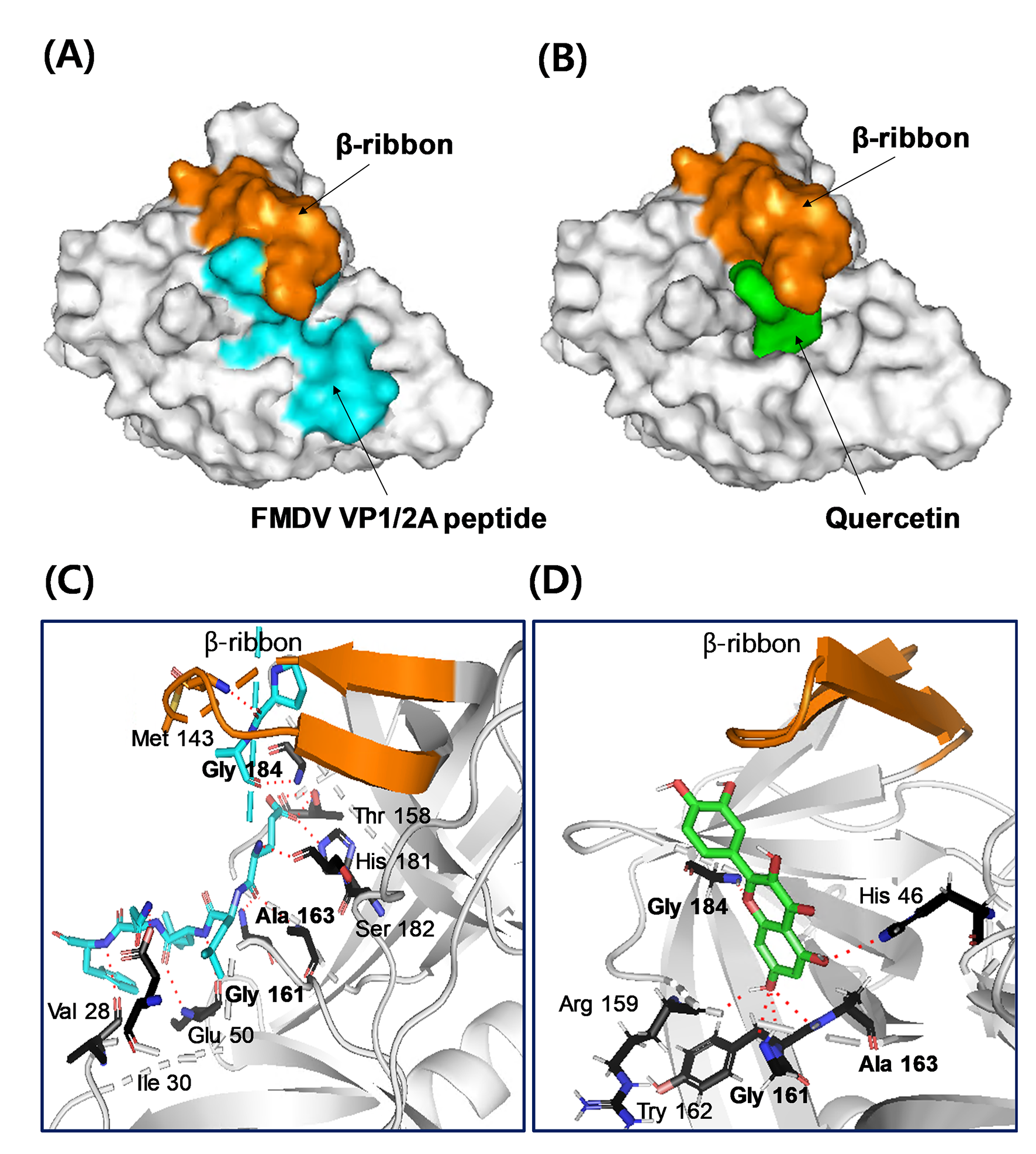

Supplement: Supplementary Figure 1 — Molecular docking prediction for evaluating the interaction between foot-and-mouth disease virus (FMDV) 3C protease and quercetin. Surface representation of the binding pocket of 3C protease (PDB ID: 2WV5) (gray) with either (A) the FMDV VP1/2A peptide (cyan) or (B) quercetin (CID: 5280343) (green), relative to the β-ribbon of 3C pro (orange), respectively. An illustration of hydrogen bond formation between FMDV 3C pro and either the FMDV VP1/2A peptide (C) or quercetin (D), depicting hydrogen bonds (dotted red lines), VP1/2A peptide residues (cyan sticks), 3C pro residues (black sticks), and quercetin residues (green sticks). Amino acids in bold are residues forming hydrogen bonds with both (C,D). [file Image_1.TIF]
